# Facility-dependent metabolic phenotype and gut bacterial composition in CD-1 mice from a single vendor: A brief report

**DOI:** 10.1371/journal.pone.0238893

**Published:** 2020-09-21

**Authors:** Allison L. Unger, Korin Eckstrom, Thomas L. Jetton, Jana Kraft

**Affiliations:** 1 Department of Animal and Veterinary Sciences, The University of Vermont, Burlington, Vermont, United States of America; 2 Department of Microbiology and Molecular Genetics, The University of Vermont, Burlington, Vermont, United States of America; 3 Division of Endocrinology, Metabolism and Diabetes, Department of Medicine, The University of Vermont, Colchester, Vermont, United States of America; Max Delbruck Centrum fur Molekulare Medizin Berlin Buch, GERMANY

## Abstract

Utilization of murine models remains a valuable tool in biomedical research, yet, disease phenotype of mice across studies can vary considerably. With advances in next generation sequencing, it is increasingly recognized that inconsistencies in host phenotype can be attributed, at least in part, to differences in gut bacterial composition. Research with inbred murine strains demonstrates that housing conditions play a significant role in variations of gut bacterial composition, however, few studies have assessed whether observed variation influences host phenotype in response to an intervention. Our study initially sought to examine the effects of a long-term (9-months) dietary intervention (*i*.*e*., diets with distinct fatty acid compositions) on the metabolic health, in particular glucose homeostasis, of genetically-outbred male and female CD-1 mice. Yet, mice were shipped from two different husbandry facilities of the same commercial vendor (Cohort A and B, respectively), and we observed throughout the study that diet, sex, and aging differentially influenced the metabolic phenotype of mice depending on their husbandry facility of origin. Examination of the colonic bacteria of mice revealed distinct bacterial compositions, including 23 differentially abundant genera and an enhanced alpha diversity in mice of Cohort B compared to Cohort A. We also observed that a distinct metabolic phenotype was linked with these differentially abundant bacteria and indices of alpha diversity. Our findings support that metabolic phenotypic variation of mice of the same strain but shipped from different husbandry facilities may be influenced by their colonic bacterial community structure. Our work is an important precautionary note for future research of metabolic diseases via mouse models, particularly those that seek to examine factors such diet, sex, and aging.

## Introduction

Microbiome structure and composition within the gastrointestinal tract have been identified as an important determinant of host disease development and severity [1–5]. Accordingly, defining the underlying mechanisms involved in the relationship between gut microbiota and host metabolic function has become an essential focus of biomedical research. Of particular interest are the bacteria, which dominate the colonization of the gastrointestinal tract compared to eukarya and archaea [6]. Yet, the role of host-related factors such as genetic background, sex, age, and lifestyle (*e*.*g*., diet) on gut bacterial composition and, thus, disease risk remains poorly understood.

The utilization of murine models is ideal to characterize the dynamics of gut bacteria and their host while minimizing the influence of confounding factors. Yet, while there are many benefits to murine models in biomedical research, reproducibility of results and variation in phenotype remains a concern [7]. Recently, it has been theorized that the observed heterogeneity in disease phenotype of mice across studies is, at least, in part due to pervasive differences in gut bacterial composition [8]. Indeed, evidence shows that the gut bacteria of mice, even within the same strain, can vary due to factors such as commercial vendor [8–12], facility [10, 11, 13, 14], room [15], and cage [16, 17]. Importantly, few studies which reported variability among the gut bacteria of mice from different vendors or facilities further assessed whether, and to what degree, these differences influenced host disease phenotype in response to an intervention. Moreover, to our knowledge, these studies have only been performed in inbred strains [8, 10–13, 15–17], while none have examined this phenomenon in the context of a genetically-heterogenous outbred mouse stock.

We originally designed an experiment to examine the effect of long-term consumption of diets varying in fat quality on metabolic health during aging. Accordingly, four experimental diets with distinct fatty acid (FA) compositions were formulated for long-term feeding: a control fat blend, based on the diet of the average U.S. American, or the control fat blend supplemented with fish oil, butter oil, or echium oil. Inadvertently, the vendor shipped outbred CD-1 mice from two different husbandry facilities two weeks apart. During the course of the study, we observed that the metabolic phenotype varied among mice from the different husbandry facilities. We hypothesized that differences in the colonic bacterial composition of mice between facilities was a contributory factor to the metabolic phenotype observed in response to the dietary intervention. To test this hypothesis, our objectives were to 1) evaluate the metabolic response to a long-term dietary intervention of male and female mice derived from different husbandry facilities, 2) evaluate variations in colonic bacterial composition of male and female mice from these facilities, and 3) assess the influence of colonic bacterial composition on the metabolic phenotype of mice from each facility.

## Methods

### Animals and experimental design

All animal procedures were approved by and conformed to the guidelines and regulations of The University of Vermont Institutional Animal Care and Use Committee. A total of 162 CD-1 mice were used (IGS #022, Charles River, Wilmington, MA, USA) with 81 mice from either the Kingston, NY (Cohort A) or Raleigh, NC (Cohort B) husbandry facility (n = 10-11/cohort/sex/diet). At three weeks of age mice arrived from each facility two weeks apart. Upon arrival, mice were randomly paired (same sex) in ventilated cages (Thorens Caging Systems, Hazelton, PA, USA). For the duration of the study, mice were housed at a temperature of 23.6°C with 64% humidity on a 12 hr light-dark cycle. During the one-week acclimation period, mice had free access to water and a standard chow of 26% protein, 60% carbohydrate, and 14% fat (Prolab^®^ Rat/Mouse/Hamster 3000, LabDiet, St. Louis, MO, USA). Mice were then fed one of four experimental high-fat (40% of total energy) diets from one until 10 months of age: 1) CO: 100% control fat; 2) FO: 70% CO supplemented with 30% fish oil; 3) BO: 70% CO supplemented with 30% butter oil; or 4) EO: 70% CO supplemented with 30% echium oil. Oil derived from fish, butter, and echium were chosen to create experimental diets with distinct fatty acid profiles (FO, BO, and EO, respectively) from one another and the CO diet. In particular, the CO diet was marked by the combination of its high content of saturated fatty acids and its high n-6/n-3 fatty acid ratio; the FO diet by its high content of long-chain n-3 fatty acids, the BO diet by its high content of short-, medium-, odd-chain, and branched-chain fatty acids; and the EO diet by its high content of n-3 fatty acids and γ-linolenic acid (n-6 fatty acid). A detailed description of the experimental diets has been previously published by Unger et al. [18].

As expected with a long-term study, there were a few (5 in total) mice that died or were euthanized before the projected completion of the study due to one of the following reasons: spinal injury (n = 1), atrial thrombosis (n = 1), fatal fight wounds (n = 2), and an unknown cause (n = 1).

### Measurement of metabolic parameters

Feed intake per cage was measured weekly. Body weight was recorded weekly for the initial three months and then monthly for the remaining duration of the study. On a monthly basis, mice were tail-nicked to measure glucose during the fed state (~9:00 AM) from whole blood with a glucose meter (Free Style Lite, Abbott, Abbott Park, IL, USA). Whole blood was collected in heparinized microhematocrit capillary tubes (Fisher Scientific, Waltham, MA, USA) and centrifuged (Sorvall^®^ Biofuge Fresco, Kendro Laboratory Products, Asheville, NC, USA) at 9,500 *g* for 5 min at 4°C; plasma was stored at -20°C until measurement of insulin concentration with a commercially available kit (Mouse Ultrasensitive Insulin ELISA, Alpco, Salem, NH, USA).

Every three months, parameters of glucose homeostasis were assessed via intraperitoneal (IP) glucose tolerance test and insulin tolerance test (GTT and ITT, respectively). IP administration of glucose and insulin was chosen over oral administration, as it is a highly reproducible method with relatively stress-free administration and is more practical for large animal cohorts. On the day of the GTT, mice were fasted for six hours before IP administration of glucose (2 g/kg; Sigma-Aldrich, St. Louis, MO, USA), and glucose from whole blood was measured at 0, 15, 30, 60, 90, and 120 min. Whole blood was also collected via tail nick at 0 and 30 min and processed as described above for determination of glucose-induced insulin secretion. Insulin resistance was assessed via the homeostatic model assessment of insulin resistance (HOMA-IR), calculated as glucose_0 min_*insulin_0 min_)/405 [19]. β-cell function was evaluated via the insulinogenic index (Δinsulin_1-30 min_)/(Δglucose_1-30 min_) [20]. Two weeks after the GTT, ITT was performed. Mice were fasted for six hours before IP administration of insulin (Humulin^®^ R (U-100); 0.75 U/kg; Eli Lilly and Company, Indianapolis, IN, USA). Over a time-course of one hour, glucose was measured at 0, 15, 30, and 60 min. Fasting insulin was measured from plasma processed from whole blood as described above.

### Analysis of colonic bacteria

Sample collection and analysis of colonic bacteria has been described previously [18]. At approximately ten months of age, following the final GTT and ITT, 24-hour fecal collection (n = 2-5/diet/sex, n = 28-31/cohort) was executed via individual metabolism cages (Techniplast, West Chester, PA, USA). Total microbial DNA was extracted and the V1-3 region of the bacterial 16S rRNA was amplified on the GeneAmp PCR System 9700 (Applied Biosystems, Foster City, CA, USA) using the primer pair 27F and 519R (Integrated DNA Technologies, Skokie, IL, USA). Amplicons were sent to Molecular Research DNA (Shallowater, TX, USA) for sequencing via Illumina Miseq (v.3). Colonic bacterial density was determined as reported in Unger et al. [18].

### Statistical analysis

Metabolic parameters were analyzed with a linear mixed model (MIXED function) with an unstructured covariance in IBM SPSS Statistics for Macintosh, Version 25.0 (IBM Corp., Armonk, NY, USA). Facility, sex, diet, and age were included in the model as fixed effects, with cage number as a random effect and body weight as a covariate. For post hoc testing, the COMPARE function was used to assess unadjusted pairwise comparisons due to the large quantity of interactions within the model. Significance was determined at *P* < 0.05. For each dependent variable, the residuals were assessed for normal distribution via Q-Q plots. The following dependent variables were transformed as follows: feed efficiency, body weight gain, and fed glucose via square root; body weight, fed insulin, fasted glucose and insulin, GTT and ITT area under the curve (AUC), insulinogenic index, and HOMA-IR via log. Bacterial density was analyzed via three-way ANOVA (including the effects of cohort, sex, and diet) in JMP^®^ (Version 14, SAS Institute Inc., Cary, NC, USA). Dependent variables with residuals that were normally distributed were not transformed. Tables and figures display non-transformed data.

### Bioinformatics analysis

Sequencing files are accessible via the Sequence Read Archive (SRA) under the accession number PRJNA484010. Paired-end sequencing was performed and OTUs were generated using the MRDNA pipeline consisting of usearch, uchime, and taxonomic classification using BLASTn top hit. The mean sequencing depth was 28,104 reads, with a minimum of 8,473 and a maximum of 76,653 reads.

Normalization, abundance, and diversity analyses were conducted in R version 3.5.1. Raw OTU counts were normalized by sample, visualized via ordination, and alpha diversity metrics (Richness, Fisher alpha, Shannon, and Pielou’s Evenness) calculated using ampvis2 [21]. Cohort and sex level ordination is shown using PCA after Hellinger transformation and using Euclidean distances. For relative abundance between cohorts at the genus level, counts were aggregated and any taxa <0.01% relative abundance were grouped into a single category named accordingly. Alpha diversity differences between cohort and sex were assessed via ANOVA using the rstatix package [22].

Differential abundance of taxa was calculated using *DESeq2* [23] at the genus level with the design of ~0+CohortSex, a feature created to collapse the effects of cohort and sex (*i*.*e*., males in Cohort A vs males in Cohort B), and an FDR cutoff of <0.05 as determined by Benjamini-Hochberg correction. The effect of diet was not considered in this model in order to increase statistical power, as inclusion of this feature resulted in one group of n = 2 (CO-fed males in Cohort B). An adjusted *P*-value <0.05 and log_2_ fold change >|1.5| was considered significant. Abundance of significant taxa were then correlated using the rstatix package. Spearman’s rank correlations were used with a significance threshold of *P*<0.05 and visualized using the cor_mat function.

## Results

### Parameters of metabolic health

**Fig 1:**
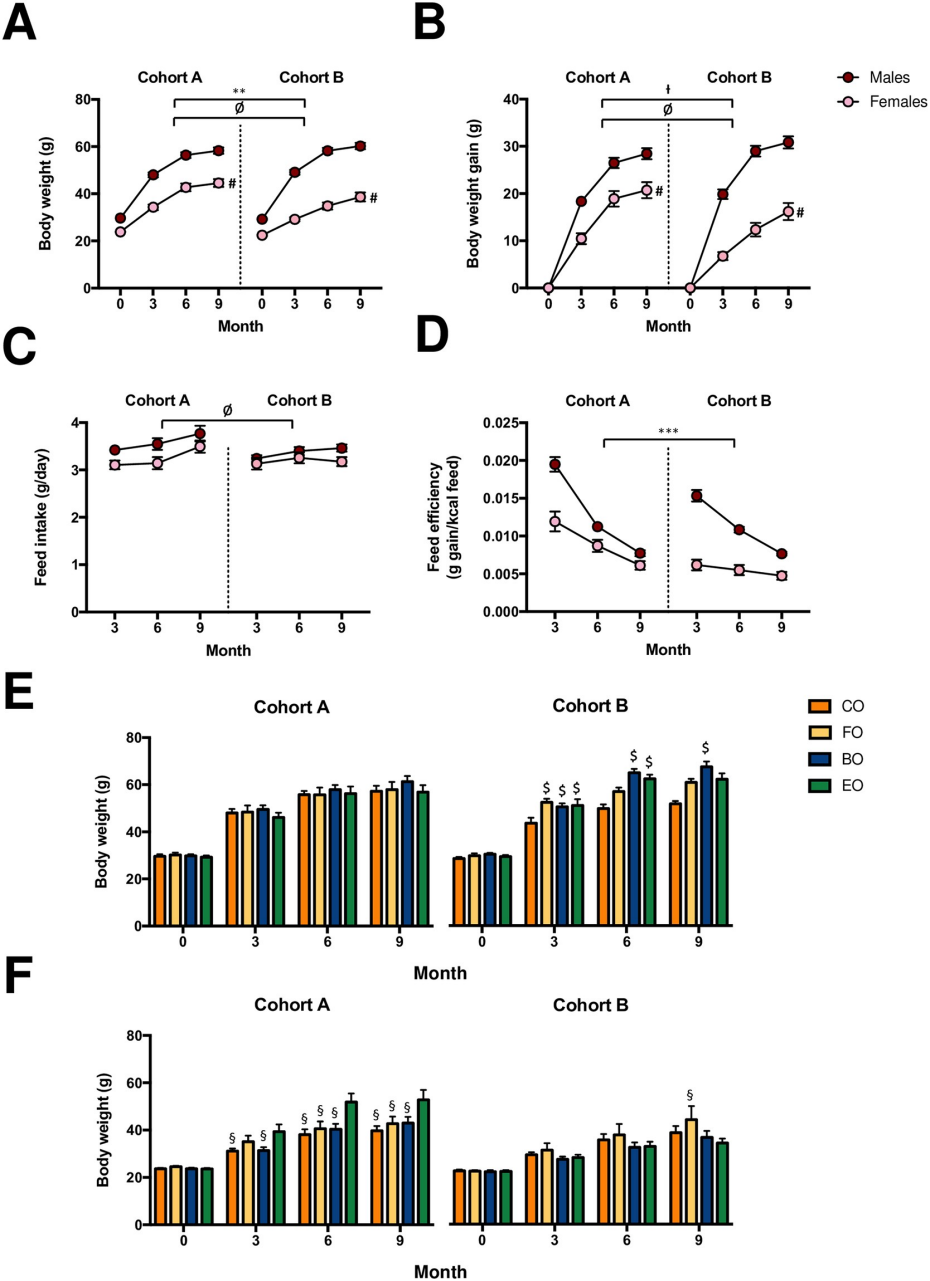
(A) Body weight, (B) weight gain, (C) feed intake, and (D) feed efficiency of male and female CD-1 mice acquired from two different animal husbandry facilities (Cohorts A and B, respectively). Body weight is displayed for (E) male and (F) female CD-1 mice fed a high-fat diet either consisting of 100% control fat (CO) or CO fat supplemented with 30% of fish oil (FO), butter oil (BO) or echium oil (EO). Mice of each cohort were allocated to one of four diet groups at four weeks of age (n = 10-11/cohort/sex/diet). Values are expressed as mean ± standard error of the mean. ** = *P* < 0.01, Cohort A vs. Cohort B collapsed by time, sex, and diet. *** = *P* < 0.001, Cohort A vs. Cohort B collapsed by time, sex, and diet. ^Ɨ^ = *P* = 0.067, Cohort A vs. Cohort B collapsed by time, sex, and diet. ^Ø^ = *P* < 0.05, males vs. females collapsed by cohort, time, and diet. ^#^ = *P* < 0.05, females of Cohort A vs. females of Cohort B collapsed by time and diet. ^$^ = *P* < 0.05, FO-, BO-, or EO-fed males vs. CO-fed males within each respective month and cohort. ^§^ = *P* < 0.05, CO-, FO-, or BO-fed females vs. EO-fed females within each respective month and cohort.

Data displaying the metabolic parameters of mice and the effects of cohort (A and B), sex (male and female), diet (CO: control diet, FO: CO supplemented with fish oil, BO: CO supplemented with butter oil, and EO: CO supplemented with echium oil), and age (3, 6, 9 months of intervention) are shown in the Supplemental Information (S1-S4 Tables of S1 File, respectively). Body weight of mice was greater (4%) at baseline in Cohort A compared to Cohort B, and the difference remained during the entirety of the study (5, 6, and 3% greater after 3, 6, and 9 months, respectively; Fig 1A; *P* < 0.05). Particularly, body weight (Fig 1A; *P* < 0.05) and weight gain (Fig 1B; *P* < 0.05) differed among females within each cohort. While a difference across cohorts was not observed for feed intake (Fig 1C), feed efficiency was 38% greater in Cohort A than in Cohort B (Fig 1D; *P* < 0.001). Notably, there was also an interactive effect of cohort, sex, diet and age on body weight. In Cohort B, FO-, BO-, and EO-fed males had a greater body weight (20, 19, and 16%, respectively) than CO-fed males after three months (*P* < 0.05), and BO-fed males sustained this greater body weight compared to CO-fed males as mice aged (31 and 30% greater after 6 and 9 months, respectively; Fig 1E; *P* < 0.05). Yet, no diet-induced differences in body weight were observed in males of Cohort A (Fig 1E). In contrast, no variations among diet groups in body weight were observed in females of Cohort B until nine months, whereas body weight was greater in EO-fed females than in other diet groups after three months (Fig 1F; *P* < 0.05).

**Fig 2:**
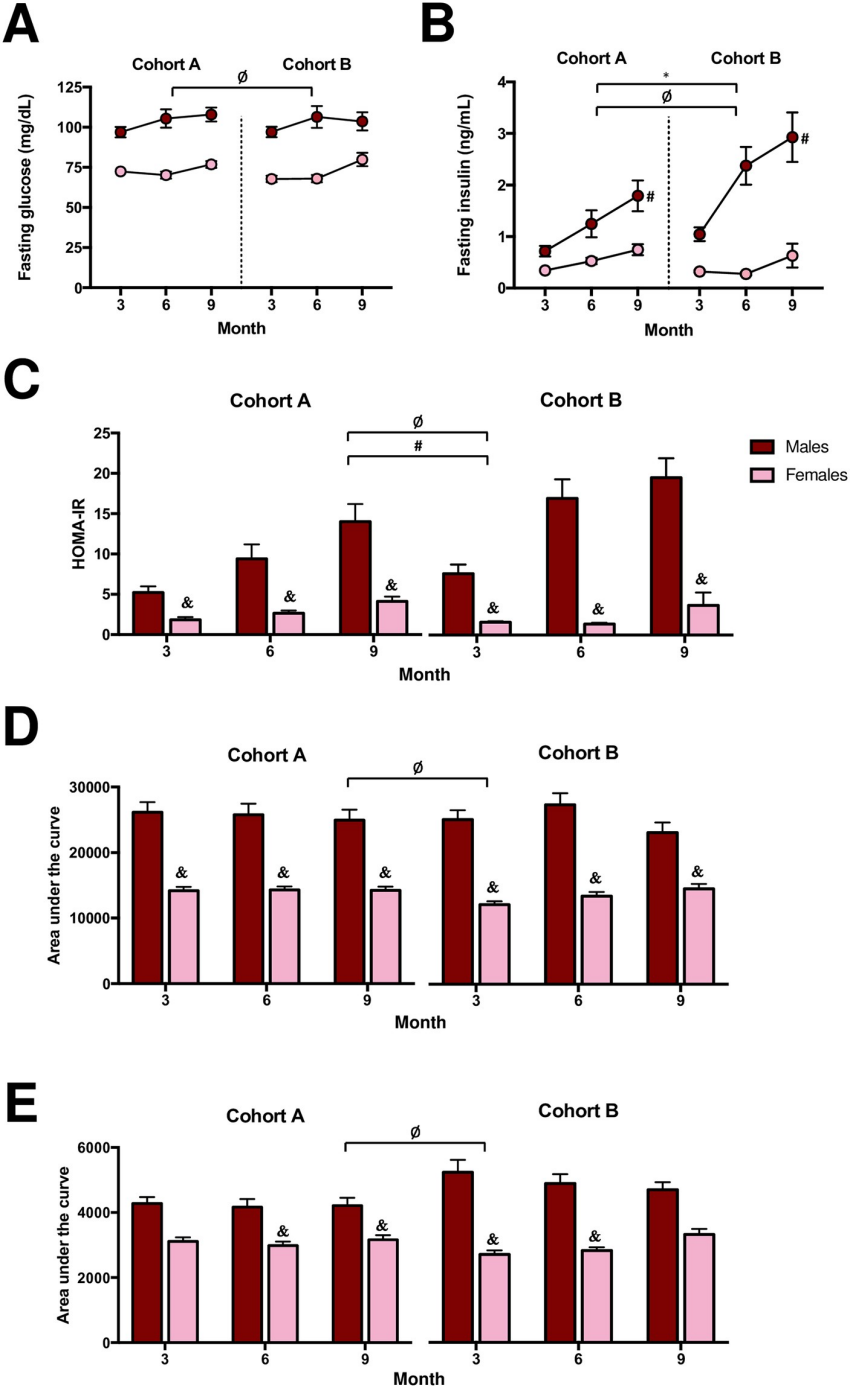
(A) Fasting glucose, (B) fasting insulin, (C) homeostatic model assessment of insulin resistance (HOMA-IR), (D) area under the curve of glucose tolerance test, and (E) area under the curve of insulin tolerance test of CD-1 mice received from two different animal husbandry facilities (Cohort A and B, respectively). Mice of each cohort were allocated to one of four diet groups at four weeks of age (n = 10-11/cohort/sex/diet). Values are expressed as mean ± standard error of the mean. * = *P* < 0.05, Cohort A vs. Cohort B collapsed by time, sex, and diet. ^Ø^ = *P* < 0.05, males vs. females collapsed by cohort, time, and diet. ^#^ = *P* < 0.05, males of Cohort A vs. males of Cohort B collapsed by time and diet. ^&^ = *P* < 0.05, males vs. females within each respective month and cohort collapsed by diet.

Fasting glucose (Fig 2A) did not vary in mice by cohort, yet, mice in Cohort B exhibited 56% higher fasting insulin levels (Fig 2B) suggesting impaired insulin sensitivity, compared to mice in Cohort A (*P* < 0.05). In particular, fasted insulin levels overall were 71% higher in males of Cohort B than Cohort A (Fig 2B; *P* < 0.05). Sex-differences evaluated at three, six, and nine months were present within each cohort for certain parameters of glucose homeostasis, including insulin sensitivity via HOMA-IR (Fig 2C; *P* < 0.05) and glucose tolerance via GTT AUC (Fig 2D; *P* < 0.05). Yet, differences in males compared to females in insulin sensitivity via ITT AUC were not detectable in Cohort A until six months (Fig 2E; *P* < 0.05). Additionally, differences in ITT AUC results between sexes were observed in Cohort B at three and six months, but dissipated by nine months (*P* < 0.05). Collectively, our data demonstrate that the effects of dietary intervention, sex, and aging influenced the metabolic phenotype of two mouse cohorts in a different manner.

### Colonic bacterial composition

**Fig 3:**
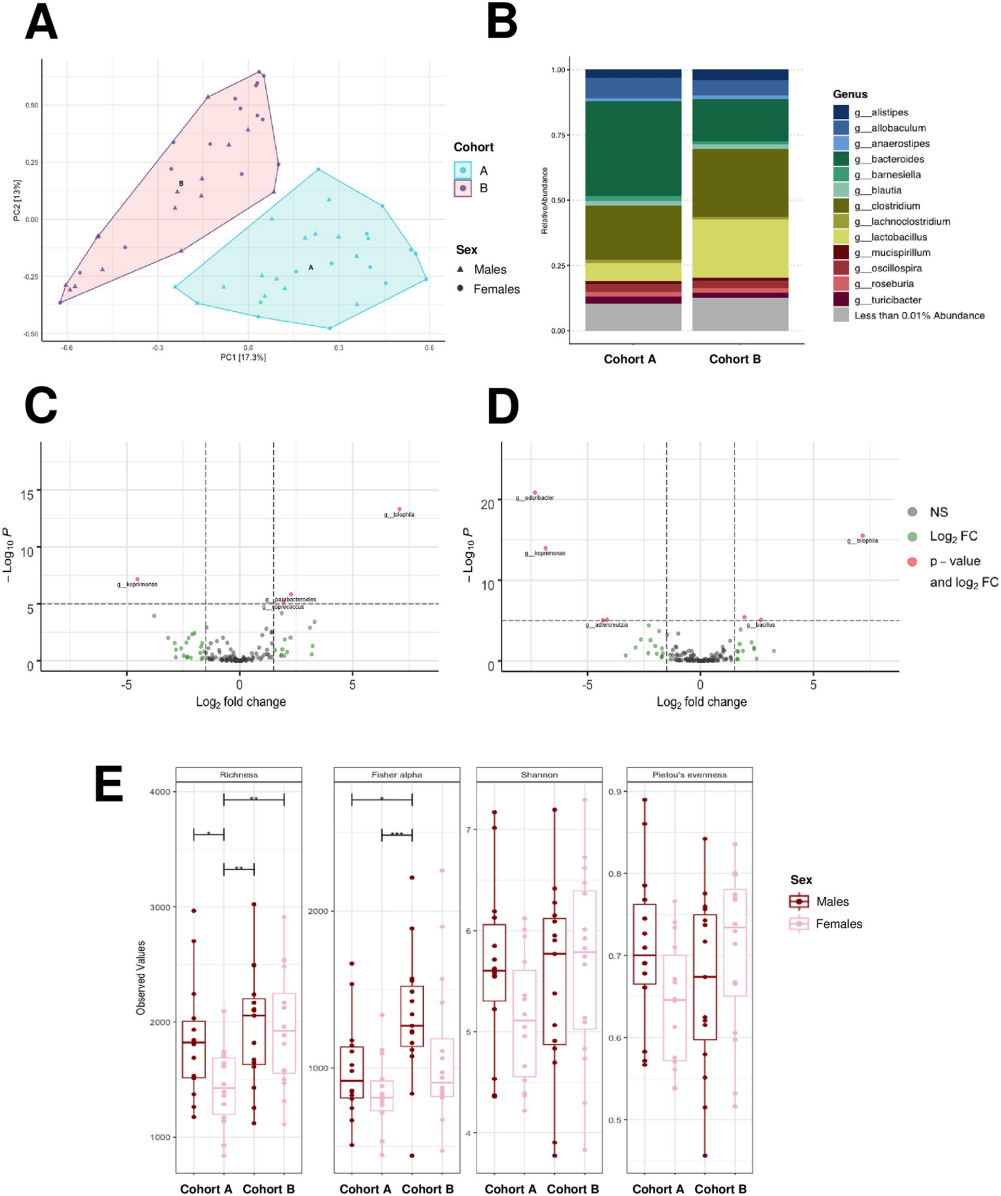
Colonic bacterial composition of male and female CD-1 mice received from two different animal husbandry facilities (Cohort A and B, respectively). (A) Principal component analysis of bacterial composition, (B) relative abundance of bacterial genera, (C) enhanced volcano plot of bacterial genera that were differentially abundant in males of Cohort A (negative x-axis) and males of Cohort B (positive x-axis), (D) enhanced volcano plot of bacterial genera that were differentially abundant in females of Cohort A (negative x-axis) and females of Cohort B (positive x-axis), and (E) alpha diversity indices (richness, Fisher’s alpha, Shannon’s index, Pielou’s evenness) of bacteria in CD-1 mice. For colonic bacterial analysis, fecal samples were collected at ten months of age from a subset of mice from each diet group (n = 2-5/diet/sex, n = 28-31/cohort). NS = not significant and < log_2_(fold change), Log_2_ FC = > log_2_(fold change), *P*-value and log_2_ FC = > log_2_(fold change) and *P* < 0.05, * = *P* < 0.05, ** = *P* < 0.01, *** = *P* < 0.001.

Principal component analysis revealed distinct differences in colonic bacterial composition of mice between the two cohorts (Fig 3A). At the genus level, 23 bacterial taxa were found to be differentially abundant between Cohort A and Cohort B (S5 Table of S1 File; *P* < 0.05 calculated with Benjamini-Hochberg adjustment determined by *DESeq2*). Specifically, the abundance of *Bacteroides* was greater (log_2_(fold change) = -1.2), while *Lactobacillus* was lower (log_2_(fold change) = 1.5), in Cohort A compared to Cohort B (S5 Table of S1 File; *P* < 0.05). These differences found in bacterial taxa via *DESeq2* between cohorts correspond with changes in relative abundance of these two major genera (Fig 3B). Furthermore, bacterial genera were found to be differentially abundant between males in Cohort A versus Cohort B, as well as females in Cohort A versus Cohort B (Figs 3C and 3D, respectively; *P* < 0.05). Box and whisker plots displaying the abundance of the top 10 most abundant genera in mice by cohort, sex, and diet are shown in the Supplemental Information (S1 Fig of S1 File).

Alpha diversity of colonic bacterial genera was greater in Cohort B than in Cohort A (*P* < 0.05), although alpha diversity was similar across sex and diet groups (S2 Fig of S1 File). Notably, sex differences in richness of bacterial taxa were found within Cohort A (*P* < 0.05), but not Cohort B (Fig 3E). Overall, in females of Cohort A, alpha diversity of bacteria was enriched in EO-fed mice and diminished in CO-fed mice (S3 Fig of S1 File; *P* < 0.05). Yet, diet did not influence the alpha diversity of bacteria in females of Cohort B or males, regardless of cohort (S3 Fig of S1 File). Thus, our findings highlight distinct sex- and diet-dependent differences in the colonic bacterial abundance and diversity of mice originating from different animal husbandry facilities.

### Correlations between metabolic parameters and colonic bacterial composition

**Fig 4:**
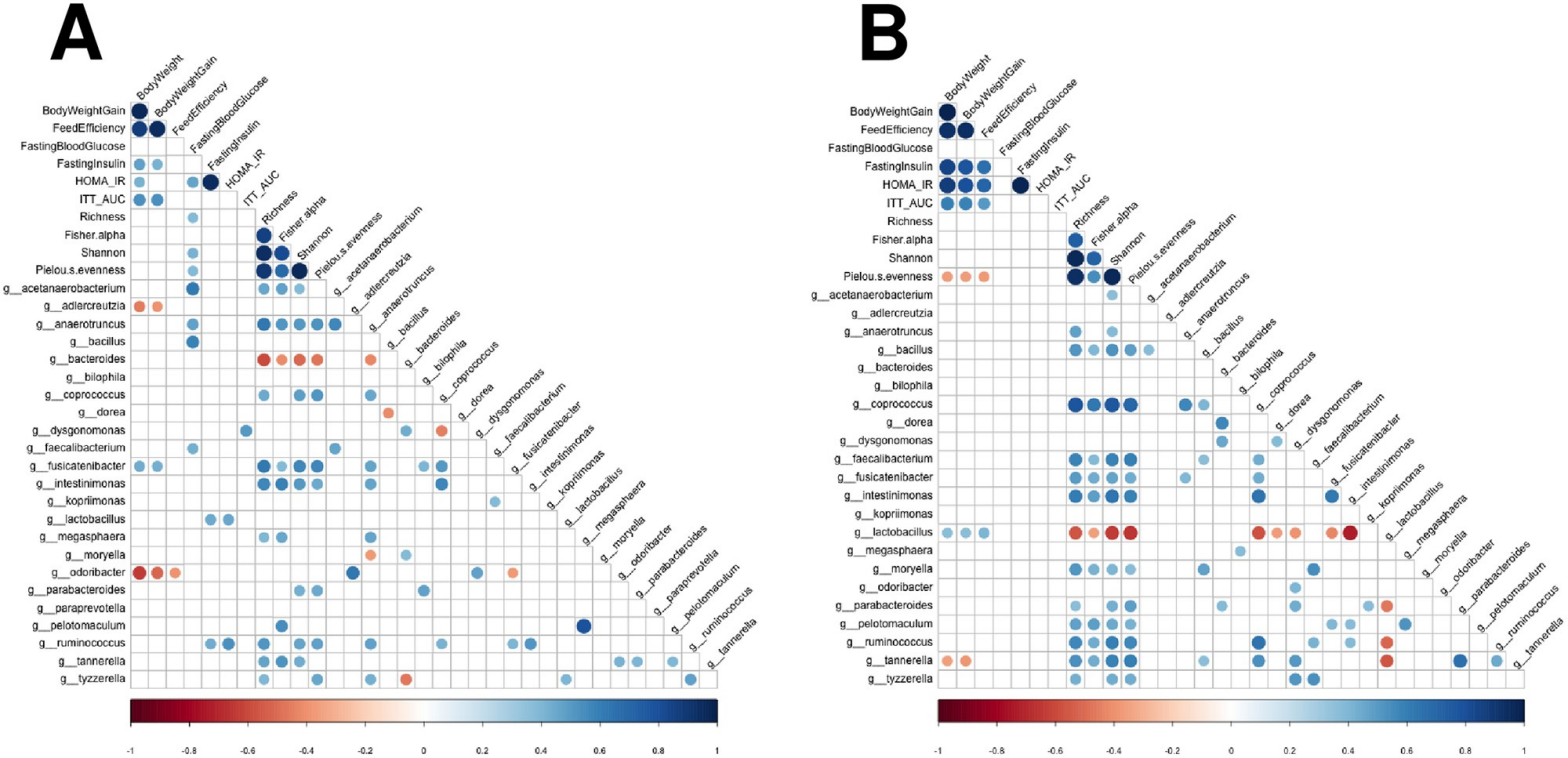
Spearman correlation matrices between metabolic parameters, alpha diversity indices of colonic bacteria, and differentially abundant colonic bacterial genera (*P* <0.05 calculated with Benjamini-Hochberg adjustment determined by *DESeq2*) of male and female mice in Cohort A (A) and Cohort B (B). For colonic bacterial analysis, fecal samples were collected at ten months of age from a subset of mice from each diet group (n = 2-5/diet/sex, n = 28-31/cohort). A positive correlation (closer to 1) is signified by a darker shade of blue; a negative correlation (closer to −1) is signified by a darker shade of red (*P* < 0.05).

Spearman correlation matrices were generated to compare the associations between metabolic parameters and colonic bacterial composition of mice in Cohort A (Fig 4A) and Cohort B (Fig 4B). Specifically, bacterial genera that were found to be differentially abundant (*P* <0.05 calculated with Benjamini-Hochberg adjustment) via *DESeq2* were evaluated. In Cohort A, *Lactobacillus* was positively correlated with fasting insulin levels (r = 0.44) and HOMA-IR (r = 0.45), indicating an association of this taxon with insulin resistance (*P* < 0.05). *Ruminococcus*, although a bacterial taxon found to be in minor relative abundance in mice (< 0.01%), was also positively associated with fasting insulin levels (r = 0.43) and HOMA-IR (r = 0.53; *P* < 0.05). Correlations were found between several other minor genera and metabolic parameters in mice of Cohort A. *Fusicatenibacter* had a moderately positive association with body weight (r = 0.43; *P* < 0.05) and weight gain (r = 0.42; *P* < 0.05). In contrast, *Adlercreutzia* and *Odoribacter* were negatively associated with body weight (r = − 0.46 and −0.64, respectively) and weight gain (r = − 0.40 and −0.52, respectively; *P* < 0.05). *Dysgonomonas* was correlated with ITT AUC (r = 0.50; *P* < 0.05), suggesting a relationship of this genus with insulin resistance. Fasting blood glucose had a strong correlation the genera *Acetanaerobacterium* (r = 0.61) and *Bacillus* (r = 0.58), suggesting a relationship between these genera and impaired glucose homeostasis. Moreover, fasting glucose was positively associated with alpha diversity indices, including richness (r = 0.38) and Shannon’s index (r = 0.41; *P* < 0.05).

Minimal correlations were found between colonic bacterial composition and metabolic parameters in mice of Cohort B (Fig 4B). *Lactobacillus* had a positive correlation with body weight, weight gain, and feed efficiency (r = 0.36, 0.36, 0.40; *P* < 0.05). In addition, alpha diversity (Pielou’s evenness) was negatively correlated with body weight (r = -0.37), weight gain (r = -0.37), and feed efficiency (r = -0.38; *P* < 0.05). Taken together, our data indicate that observed differences in metabolic phenotype of mice received from different animal husbandry facilities may be influenced by the underlying variance in their colonic bacterial compositions, including abundance and alpha diversity.

### Colonic bacterial density

Colonic bacterial density, determined as bacterial copies per μg wet fecal pellet, did not differ in mice of different sexes and diet groups, nor were any interactions among any main effects observed. Of note, bacterial density differed by cohort (14.2 ± 0.05 and 13.9 ± 0.05 log copies/μg fecal pellet in Cohort A versus Cohort B, respectively, when collapsed by sex and diet; *P* < 0.001). Thus, colonic bacterial density of mice may be an additional factor in the distinctions observed among mice of different cohorts.

## Discussion

The utilization of murine models in biomedical research is often an important and necessary step in developing a better understanding of a disease pathology, yet, there is a growing concern regarding the reproducibility of results from murine-based studies [7]. We initially designed a study to examine the effect of a dietary intervention (*i*.*e*., fat quality) on parameters of glucose homeostasis in male and female outbred CD-1 mice during aging. Importantly, while mice were inadvertently shipped from two different animal husbandry facilities of the same commercial vendor, we observed variations in diet-induced metabolic phenotype from the different facilities over the course of the study. Thus, we sought to determine the differences in colonic bacterial composition of mice from each facility (Cohort A and B, respectively) and to assess the influence of colonic bacterial composition on the metabolic phenotype of mice.

Recent work [24, 25] highlights that inconsistent phenotypes of mice in response to an intervention across vendors, even among inbred strains, continues to be a widespread issue in biomedical research. For example, Hull et al. [25] described that substrains of male C57BL/6, a commonly utilized mouse strain in diabetes research, had variable blood glucose levels and insulin response after an 18-week high-fat diet. Importantly, however, these studies did not evaluate the gut microbial composition of these substrains of mice as a potential factor [24]. However, other work confirms clear disparities in gut bacterial composition of mice, particularly by vendor [8–12,26] as well as facility [10, 11, 13, 14]. In our study, we found that colonic bacterial community structure differed between the two cohorts of mice. Moreover, colonic bacterial taxa and indices of alpha diversity correlated with disparate metabolic outcomes in mice from each cohort, exposing a strong effect of facility of origin on phenotype. In accordance with our work, Randall et al. [27] originally sought to assess the effects of an intervention (*i*.*e*., uremia on the urinary metabolome and gut microbiome) in an outbred rodent model, while instead finding that differences in the cecal bacterial abundance and alpha diversity of Wistar rats from separate shipments significantly altered experimental results. Yet, sex differences were not assessed within this context as only male rats were utilized for this study.

Diet is considered to be a primary risk factor in the pathogenesis of metabolic diseases [28–30], and in particular, changes in dietary fat quality (*i*.*e*., fatty acid composition) are shown to have an influential role in the development of metabolic impairments [31]. Research indicates that one of the avenues in which dietary fat quality likely modulates disease risk is through manipulation of the gut microbiome [18, 32–36]. However, it would be challenging to assess long-term effects of dietary fat quality and its interaction with gut microbiota during aging in humans without interference from confounding variables, thus rationalizing the use of a mouse model. Despite the original objective of our research, we found that several differentially abundant taxa were associated with parameters of weight gain and glucose homeostasis, suggesting that underlying diversity in the gut bacterial composition between cohorts had a distinct impact on systemic metabolism and overall disease risk. For example, in Cohort A, but not Cohort B, a relationship was observed between the genera *Lactobacillus* and *Ruminococcus* and insulin resistance, a key feature of type 2 diabetes pathogenesis [37–40]. Indeed, previous research has reported a greater abundance of *Ruminococcus* in individuals with prediabetes [41] and type 2 diabetes [42–44] compared to individuals with normal glucose control. Similar associations have been found regarding *Lactobacillus* [42, 45, 46]. Thus, it is conceivable that these two genera played a role in the divergent insulin sensitivity observed between the two cohorts of mice. Due to the nature of our study design and *ad-hoc* scientific inquiry, however, it is important to note that it is not possible to determine a causal effect of colonic bacterial genera on the metabolic phenotype of CD-1 mice in the present work.

Our work provides evidence that variance in commensal bacteria in mice from different facilities of the same vendor likely impact the metabolic phenotype in response to a dietary intervention. Indeed, should we have used mice from only one of these cohorts, our conclusions regarding the effects of diet, sex, and aging on metabolic health would have been misrepresented and incomplete. Specific to this work, there are important considerations to address as part of the interpretation of our findings. For example, as our study employed a genetically-diverse outbred mouse stock, genetic variation would be expected to be a contributory factor in phenotypic variation. CD-1 mice purchased for this study, however, were bred in accordance with the International Genetic Standardization (IGS) Program [47] through Charles River, and as such there is an expectation that mice from different facilities would have comparable genetic variation. Nevertheless, we cannot determine if the observed differences were due to naturally occurring genetic drift among, and even within, animal husbandry facilities over time. Another limitation is that sequencing of a mock community was not performed, and thus we cannot fully assess the potential occurrence of contamination. Importantly, any presence of sequencing bias would have impacted all samples in a similar manner, and thus our findings (*i*.*e*., differences in colonic bacteria of cohorts) would be consistent. Sequencing accuracy was also enforced both by filtering for low quality bases (<Q25) and removal of low abundance OTUs (<0.01%) that may have resulted from error throughout the extraction and sequencing process. Additionally, we did not collect fecal samples from the time of baseline to analyze differences in colonic bacterial composition between cohorts before the start of the experiment. Yet, the analysis of the colonic bacterial composition was not the original objective of our study. Future work devoted to this inquiry, which documents differences in the phenotype of mice within and across animal husbandry facilities over time (*e*.*g*., shipments of mice two weeks apart), is essential to develop a robust understanding of the scope of such observed phenotypic differences. In particular, more in-depth analysis of the microbiome (*e*.*g*., whole genome and/or whole exome sequencing) would be highly valuable for this effort. Overall, our findings serve as a precautionary warning that experimental outcomes can drastically differ in mice depending on their animal husbandry facility of origin. Future studies along these lines should consider obtaining a specific mouse strain derived from several geographically separate facilities to validate a given set of phenotypic/metabolic outcomes. Moving forward, reproducibility of work in mouse models also hinges on a rigorous commitment to collect and report relevant metadata for the benefit of all areas of research.

## Supporting information

S1 File (DOCX)
