# Supplementary material for: Facility-dependent metabolic phenotype and gut bacterial composition in CD-1 mice from a single vendor: A brief report
Source: PLoS One. 2020 Sep 21;15(9):e0238893. doi: 10.1371/journal.pone.0238893 (PMC7505418; doi:10.1371/journal.pone.0238893)
Supplement: S1 File — (DOCX) [file pone.0238893.s001.docx]

|  | Cohort | |  | *P* value | | | | | | | | | | | | | | |
| --- | --- | --- | --- | --- | --- | --- | --- | --- | --- | --- | --- | --- | --- | --- | --- | --- | --- | --- |
|  | A^1^ | B^2^ |  | C^3^ | S^4^ | D^5^ | T^6^ | S x C | D x C | C x T | S x D | S x T | D x T | S x D x C | S x C x T | D x C x T | S x D x T | S x D x C x T |
| Daily feed intake (g) | 3.4  ± 0.1 | 3.3  ± 0.0 |  | - | 0.029 | - | <0.001 | - | - | - | - | - | 0.005 | - | - | <0.001 | - | - |
| Feed efficiency^7^ | 0.011  ± 0.000^a^ | 0.008  ± 0.000^b^ |  | <0.001 | - | - | <0.001 | - | - | <0.001 | - | <0.001 | - | - | 0.016 | - | - | 0.026 |
| Body weight  (g) | 47.2  ± 0.8^a^ | 45.1  ± 0.9^b^ |  | 0.006 | <0.001 | - | <0.001 | <0.001 | - | - | - | <0.001 | 0.002 | 0.012 | 0.002 | - | - | 0.006 |
| Body weight gain (g) | 20.4  ± 0.7 | 19.2  ± 0.8 |  | 0.067 | <0.001 | - | <0.001 | <0.001 | - | - | - | 0.007 | 0.003 | 0.002 | 0.025 | - | - | 0.057 |
| Fed glucose (mg/dL) | 102  ± 2 | 102  ± 2 |  | - | 0.001 | - | 0.001 | - | - | - | - | 0.002 | 0.057 | - | 0.064 | - | - | - |
| Fasted glucose  (mg/dL) | 88  ± 2 | 87  ± 2 |  | - | <0.001 | - | 0.023 | - | - | - | - | 0.047 | - | - | - | - | - | - |
| Fed insulin  (ng/mL) | 5.18  ± 0.37 | 5.45  ± 0.40 |  | - | <0.001 | - | 0.016 | - | - | - | - | <0.001 | - | - | - | - | - | 0.05 |
| Fasted insulin  (ng/mL) | 0.90  ± 0.08^b^ | 1.40  ± 0.14^a^ |  | 0.03 | <0.001 | - | <0.001 | 0.001 | - | - | - | 0.004 | - | - | 0.004 | - | - | - |
| Area under the curve (GTT^8^) | 19989  ± 634 | 19141  ± 627 |  | - | <0.001 | - | 0.082 | - | - | - | - | 0.038 | 0.001 | - | - | - | 0.001 | - |
| Area under the curve (ITT^9^) | 3617  ± 84 | 3666  ± 111 |  | - | <0.001 | - | 0.014 | - | - | - | 0.039 | - | 0.094 | - | 0.025 | - | - | - |
| HOMA-IR^10^ | 6.24  ± 0.59 | 9.38  ± 0.88 |  | 0.09 | <0.001 | - | <0.001 | 0.004 | - | - | - | 0.011 | - | - | 0.016 | - | - | - |
| Insulinogenic index^11^ | 3.62  ± 0.38 | 4.66  ± 0.80 |  | - | 0.007 | - | <0.001 | - | 0.04 | - | - | - | 0.042 | - | - | - | 0.022 | - |

**S1 Table.** Daily feed intake, feed efficiency, body weight, and parameters of glucose homeostasis of CD-1® mice received from two different animal husbandry facilitates (Cohort A and B, respectively). Values are expressed as mean ± standard error of the mean. Means without a common letter differ (*P* < 0.05).

^1^ CD-1® mice received from Charles River animal husbandry facility in Kingston, NY. ^2^ CD-1® mice received from Charles River animal husbandry facility in Raleigh, NC.^3^ C = Cohort.^4^ S = Sex. ^5^ D = Diet. ^6^ T= Time (month of data collection). ^7^ Feed efficiency = total weight gain (g) / total feed intake (kcal). ^8^ GTT = Glucose tolerance test. ^9^ ITT = Insulin tolerance test. ^10^ HOMA-IR = Homeostatic model of assessment of insulin resistance (glucose_0 min_*insulin_0 min_) / 405. ^11^ Insulinogenic index = ($\Delta$insulin_1-30 min_)/($\Delta$glucose_1-30 min_).

**S2 Table.** Daily feed intake, feed efficiency, body weight, and parameters of glucose homeostasis of male and female CD-1® mice. Values are expressed as mean ± standard error of the mean. Means without a common letter differ (*P* < 0.05).

|  | Sex | |  | *P* value | | | | | | | | | | | | | | |
| --- | --- | --- | --- | --- | --- | --- | --- | --- | --- | --- | --- | --- | --- | --- | --- | --- | --- | --- |
|  | Male | Female |  | C^1^ | S^2^ | D^3^ | T^4^ | S x C | D x C | C x T | S x D | S x T | D x T | S x D x C | S x C x T | D x C x T | S x D x T | S x D x C x T |
| Daily feed intake (g) | 3.5  ± 0.0^a^ | 3.2  ± 0.0^b^ |  | - | 0.029 | - | <0.001 | - | - | - | - | - | 0.005 | - | - | <0.001 | - | - |
| Feed efficiency^5^ | 0.012  ± 0.000^a^ | 0.007  ± 0.000^b^ |  | <0.001 | - | - | <0.001 | - | - | <0.001 | - | <0.001 | - | - | 0.016 | - | - | 0.026 |
| Body weight  (g) | 55.0  ± 0.6^a^ | 37.4  ± 0.7^b^ |  | 0.006 | <0.001 | - | <0.001 | <0.001 | - | - | - | <0.001 | 0.002 | 0.012 | 0.002 | - | - | 0.006 |
| Body weight gain (g) | 25.4  ± 0.5^a^ | 14.2  ± 0.7^b^ |  | 0.067 | <0.001 | - | <0.001 | <0.001 | - | - | - | 0.007 | 0.003 | 0.002 | 0.025 | - | - | 0.057 |
| Fed glucose (mg/dL) | 115  ± 3^a^ | 88  ± 1^b^ |  | - | 0.001 | - | 0.001 | - | - | - | - | 0.002 | 0.057 | - | 0.064 | - | - | - |
| Fasted glucose  (mg/dL) | 103  ± 2^a^ | 73  ± 1^b^ |  | - | <0.001 | - | 0.023 | - | - | - | - | 0.047 | - | - | - | - | - | - |
| Fed insulin  (ng/mL) | 8.64  ± 0.37^a^ | 1.74  ± 0.22^b^ |  | - | <0.001 | - | 0.016 | - | - | - | - | <0.001 | - | - | - | - | - | 0.05 |
| Fasted insulin  (ng/mL) | 1.69  ± 0.13^a^ | 0.47  ± 0.04^b^ |  | 0.03 | <0.001 | - | <0.001 | 0.001 | - | - | - | 0.004 | - | - | 0.004 | - | - | - |
| Area under the curve (GTT^6^) | 25220  ± 649^a^ | 13694  ± 248^b^ |  | - | <0.001 | - | 0.082 | - | - | - | - | 0.038 | 0.001 | - | - | - | 0.001 | - |
| Area under the curve (ITT^7^) | 4510  ± 111^a^ | 2988  ± 56^b^ |  | - | <0.001 | - | 0.014 | - | - | - | 0.039 | - | 0.094 | - | 0.025 | - | - | - |
| HOMA-IR^8^ | 12.10  ± 0.83^a^ | 2.50  ± 0.26^b^ |  | 0.09 | <0.001 | - | <0.001 | 0.004 | - | - | - | 0.011 | - | - | 0.016 | - | - | - |
| Insulinogenic index^9^ | 4.29  ± 0.75^a^ | 3.96  ± 0.36^b^ |  | - | 0.007 | - | <0.001 | - | 0.04 | - | - | - | 0.042 | - | - | - | 0.022 | - |

^1^ C = Cohort.^2^ S = Sex. ^3^ D = Diet. ^4^ T= Time (month of data collection). ^5^ Feed efficiency = total weight gain (g) / total feed intake (kcal). ^6^ GTT = Glucose tolerance test. ^7^ ITT = Insulin tolerance test. ^8^ HOMA-IR = Homeostatic model of assessment of insulin resistance (glucose_0 min_*insulin_0 min_) / 405. ^9^ Insulinogenic index = ($\Delta$insulin_1-30 min_)/($\Delta$glucose_1-30 min_).

**S3 Table.** Daily feed intake, feed efficiency, body weight, and parameters of glucose homeostasis of CD-1® mice fed a high-fat diet either consisting of 100% control fat (CO) or CO fat supplemented with 30% of fish oil (FO), butter oil (BO), or echium oil (EO). Values are expressed as mean ± standard error of the mean. Means without a common letter differ (*P* < 0.05).

|  | Diet | | | |  | |  | *P* value | | | | | | | | | | | | | |
| --- | --- | --- | --- | --- | --- | --- | --- | --- | --- | --- | --- | --- | --- | --- | --- | --- | --- | --- | --- | --- | --- |
|  | CO | FO | BO | EO |  | C^1^ | | S^2^ | D^3^ | T^4^ | S x C | D x C | C x T | S x D | S x T | D x T | S x D x C | S x C x T | D x C x T | S x D x T | S x D x C x T |
| Daily feed intake (g) | 3.2  ± 0.1 | 3.3  ± 0.1 | 3.4  ± 0.1 | 3.4  ± 0.1 |  | - | | 0.029 | - | <0.001 | - | - | - | - | - | 0.005 | - | - | <0.001 | - | - |
| Feed efficiency^5^ | 0.009  ± 0.000 | 0.010  ± 0.001 | 0.010  ± 0.001 | 0.011  ± 0.001 |  | <0.001 | | - | - | <0.001 | - | - | <0.001 | - | <0.001 | - | - | 0.016 | - | - | 0.026 |
| Body weight  (g) | 43.2  ± 1.0 | 46.7  ± 1.2 | 46.7  ± 1.3 | 48.1  ± 1.2 |  | 0.006 | | <0.001 | - | <0.001 | <0.001 | - | - | - | <0.001 | 0.002 | 0.012 | 0.002 | - | - | 0.006 |
| Body weight gain (g) | 17.1  ± 0.8 | 20.1  ± 1.0 | 20.2  ± 1.1 | 21.9  ± 1.1 |  | 0.067 | | <0.001 | - | <0.001 | <0.001 | - | - | - | 0.007 | 0.003 | 0.002 | 0.025 | - | - | 0.057 |
| Fed glucose (mg/dL) | 102  ± 3 | 99  ± 3 | 105  ± 3 | 102  ± 3 |  | - | | 0.001 | - | 0.001 | - | - | - | - | 0.002 | 0.057 | - | 0.064 | - | - | - |
| Fasted glucose  (mg/dL) | 88  ± 2 | 85  ± 2 | 92  ± 3 | 87  ± 3 |  | - | | <0.001 | - | 0.023 | - | - | - | - | 0.047 | - | - | - | - | - | - |
| Fed insulin  (ng/mL) | 4.23  ± 0.49 | 5.39  ± 0.56 | 5.66  ± 0.56 | 5.95  ± 0.6 |  | - | | <0.001 | - | 0.016 | - | - | - | - | <0.001 | - | - | - | - | - | 0.05 |
| Fasted insulin  (ng/mL) | 1.08  ± 0.15 | 0.98  ± 0.13 | 1.22  ± 0.19 | 1.27  ± 0.17 |  | 0.03 | | <0.001 | - | <0.001 | 0.001 | - | - | - | 0.004 | - | - | 0.004 | - | - | - |
| Area under the curve (GTT^6^) | 19222  ± 863 | 18935  ± 779 | 20386  ± 1050 | 19633  ± 856 |  | - | | <0.001 | - | 0.082 | - | - | - | - | 0.038 | 0.001 | - | - | - | 0.001 | - |
| Area under the curve (ITT^7^) | 3538  ± 136 | 3675  ± 123 | 3767  ± 176 | 3579  ± 106 |  | - | | <0.001 | - | 0.014 | - | - | - | 0.039 | - | 0.094 | - | 0.025 | - | - | - |
| HOMA-IR^8^ | 7.61  ± 1.15 | 6.09  ± 0.76 | 8.98  ± 1.17 | 8.17  ± 1.02 |  | 0.09 | | <0.001 | - | <0.001 | 0.004 | - | - | - | 0.011 | - | - | 0.016 | - | - | - |
| Insulinogenic index^9^ | 3.90  ± 0.94 | 4.20  ± 0.52 | 3.13  ± 0.60 | 5.30  ± 1.22 |  | - | | 0.007 | - | <0.001 | - | 0.04 | - | - | - | 0.042 | - | - | - | 0.022 | - |

^1^ C = Cohort.^2^ S = Sex. ^3^ D = Diet. ^4^ T= Time (month of data collection). ^5^ Feed efficiency = total weight gain (g) / total feed intake (kcal). ^6^ GTT = Glucose tolerance test. ^7^ ITT = Insulin tolerance test. ^8^ HOMA-IR = Homeostatic model of assessment of insulin resistance (glucose_0 min_*insulin_0 min_) / 405. ^9^ Insulinogenic index = ($\Delta$insulin_1-30 min_)/($\Delta$glucose_1-30 min_).

**S4 Table.** Daily feed intake, feed efficiency, body weight, and parameters of glucose homeostasis of CD-1® mice at three, six, and nine months of consuming a high-fat experimental diet. Values are expressed as mean ± standard error of the mean. Means without a common letter differ (*P* < 0.05).

|  | Month | | |  | *P* value | | | | | | | | | | | | | | |
| --- | --- | --- | --- | --- | --- | --- | --- | --- | --- | --- | --- | --- | --- | --- | --- | --- | --- | --- | --- |
|  | 3 | 6 | 9 |  | C^1^ | S^2^ | D^3^ | T^4^ | S x C | D x C | C x T | S x D | S x T | D x T | S x D x C | S x C x T | D x C x T | S x D x T | S x D x C x T |
| Daily feed intake (g) | 3.2  ± 0.0^b^ | 3.3  ± 0.1^a^ | 3.5  ± 0.1^a^ |  | - | 0.029 | - | <0.001 | - | - | - | - | - | 0.005 | - | - | <0.001 | - | - |
| Feed efficiency^5^ | 0.013  ± 0.001 | 0.009  ± 0.000 | 0.006  ± 0.000 |  | <0.001 | - | - | <0.001 | - | - | <0.001 | - | <0.001 | - | - | 0.016 | - | - | 0.026 |
| Body weight  (g) | 40.2  ± 0.9^c^ | 48.1  ± 1.0^b^ | 50.3  ± 1.1^a^ |  | 0.006 | <0.001 | - | <0.001 | <0.001 | - | - | - | <0.001 | 0.002 | 0.012 | 0.002 | - | - | 0.006 |
| Body weight gain (g) | 13.9  ± 0.7^c^ | 21.7  ± 0.9^b^ | 24.0  ± 0.9^a^ |  | 0.067 | <0.001 | - | <0.001 | <0.001 | - | - | - | 0.007 | 0.003 | 0.002 | 0.025 | - | - | 0.057 |
| Fed glucose (mg/dL) | 101  ± 2^a^ | 107  ± 3^a^ | 98  ± 2^b^ |  | - | 0.001 | - | 0.001 | - | - | - | - | 0.002 | 0.057 | - | 0.064 | - | - | - |
| Fasted glucose  (mg/dL) | 84  ± 2^b^ | 87  ± 3^b^ | 92  ± 2^a^ |  | - | <0.001 | - | 0.023 | - | - | - | - | 0.047 | - | - | - | - | - | - |
| Fed insulin  (ng/mL) | 3.33  ± 0.30^b^ | 5.06  ± 0.38^a^ | 7.70  ± 0.62^a^ |  | - | <0.001 | - | 0.016 | - | - | - | - | <0.001 | - | - | - | - | - | 0.05 |
| Fasted insulin  (ng/mL) | 0.64  ± 0.06^b^ | 1.13  ± 0.13^b^ | 1.67  ± 0.19 ^a^ |  | 0.03 | <0.001 | - | <0.001 | 0.001 | - | - | - | 0.004 | - | - | 0.004 | - | - | - |
| Area under the curve (GTT^6^) | 19139  ± 763 | 20302  ± 837 | 19182  ± 714 |  | - | <0.001 | - | 0.082 | - | - | - | - | 0.038 | 0.001 | - | - | - | 0.001 | - |
| Area under the curve (ITT^7^) | 3721  ± 135^a^ | 3489  ± 117^b^ | 3699  ± 108^b^ |  | - | <0.001 | - | 0.014 | - | - | - | 0.039 | - | 0.094 | - | 0.025 | - | - | - |
| HOMA-IR^8^ | 4.37  ± 0.45^b^ | 7.72  ± 0.91^b^ | 11.29  ± 1.15^a^ |  | 0.09 | <0.001 | - | <0.001 | 0.004 | - | - | - | 0.011 | - | - | 0.016 | - | - | - |
| Insulinogenic index^9^ | 2.92  ± 0.75^b^ | 2.45  ± 0.30^c^ | 7.30  ± 1.08^a^ |  | - | 0.007 | - | <0.001 | - | 0.04 | - | - | - | 0.042 | - | - | - | 0.022 | - |

^1^ C = Cohort.^2^ S = Sex. ^3^ D = Diet. ^4^ T= Time (month of data collection). ^5^ Feed efficiency = total weight gain (g) / total feed intake (kcal). ^6^ GTT = Glucose tolerance test. ^7^ ITT = Insulin tolerance test. ^8^ HOMA-IR = Homeostatic model of assessment of insulin resistance (glucose_0 min_*insulin_0 min_) / 405. ^9^ Insulinogenic index = ($\Delta$insulin_1-30 min_)/($\Delta$glucose_1-30 min_).

**S5 Table.** Results of *DEseq2* displaying differentially abundant (*P* < 0.05) bacterial genera between CD-1® mice received from two different animal husbandry facilities (Cohort A and B, respectively).

| Genus | Base Mean^1^ | Log_2_ Fold Change^2^ | SE^3^ | Stat^4^ | *P* value | Adjusted *P* value^5^ |
| --- | --- | --- | --- | --- | --- | --- |
| *Acetanaerobacterium* | 33.661 | 0.870 | 0.318 | 2.741 | 0.006 | 0.031 |
| *Adlercreutzia* | 1.687 | -4.082 | 0.677 | -6.027 | 0.000 | 0.000 |
| *Anaerotruncus* | 22.259 | -1.346 | 0.338 | -3.978 | 0.000 | 0.001 |
| *Bacillus* | 3.131 | 1.812 | 0.390 | 4.643 | 0.000 | 0.000 |
| *Bacteroides* | 6642.494 | -1.228 | 0.368 | -3.334 | 0.001 | 0.006 |
| *Bilophila* | 102.663 | 7.142 | 0.639 | 11.170 | 0.000 | 0.000 |
| *Coprococcus* | 55.178 | 1.942 | 0.297 | 6.545 | 0.000 | 0.000 |
| *Dorea* | 121.853 | -1.583 | 0.555 | -2.851 | 0.004 | 0.025 |
| *Dysgonomonas* | 2.895 | 2.091 | 0.639 | 3.274 | 0.001 | 0.007 |
| *Faecalibacterium* | 1.015 | 1.498 | 0.585 | 2.562 | 0.010 | 0.046 |
| *Fusicatenibacter* | 4.197 | -1.259 | 0.412 | -3.052 | 0.002 | 0.014 |
| *Intestinimonas* | 133.250 | -0.857 | 0.199 | -4.311 | 0.000 | 0.000 |
| *Kopriimonas* | 30.943 | -5.648 | 0.612 | -9.229 | 0.000 | 0.000 |
| *Lactobacillus* | 4525.116 | 1.522 | 0.565 | 2.692 | 0.007 | 0.033 |
| *Megasphaera* | 2.081 | -3.103 | 0.880 | -3.526 | 0.000 | 0.003 |
| *Moryella* | 0.711 | 2.139 | 0.770 | 2.780 | 0.005 | 0.029 |
| *Odoribacter* | 164.341 | -3.481 | 0.632 | -5.504 | 0.000 | 0.000 |
| *Parabacteroides* | 441.781 | 1.957 | 0.342 | 5.719 | 0.000 | 0.000 |
| *Paraprevotella* | 0.478 | -2.458 | 0.975 | -2.521 | 0.012 | 0.049 |
| *Pelotomaculum* | 1.127 | -2.368 | 0.869 | -2.726 | 0.006 | 0.031 |
| *Ruminococcus* | 202.629 | 1.381 | 0.322 | 4.290 | 0.000 | 0.000 |
| *Tannerella* | 6.484 | -1.837 | 0.424 | -4.331 | 0.000 | 0.000 |
| *Tyzzerella* | 69.071 | -1.212 | 0.308 | -3.938 | 0.000 | 0.001 |

^1^Mean of normalized counts for all samples. ^2^Effect size estimate. ^3^Standard error for log_2_(fold change). ^4^Log_2_(fold change) divided by its standard error. ^5^*P* value calculated with Benjamini-Hochberg adjustment.

**A**

**B**

**C=C**


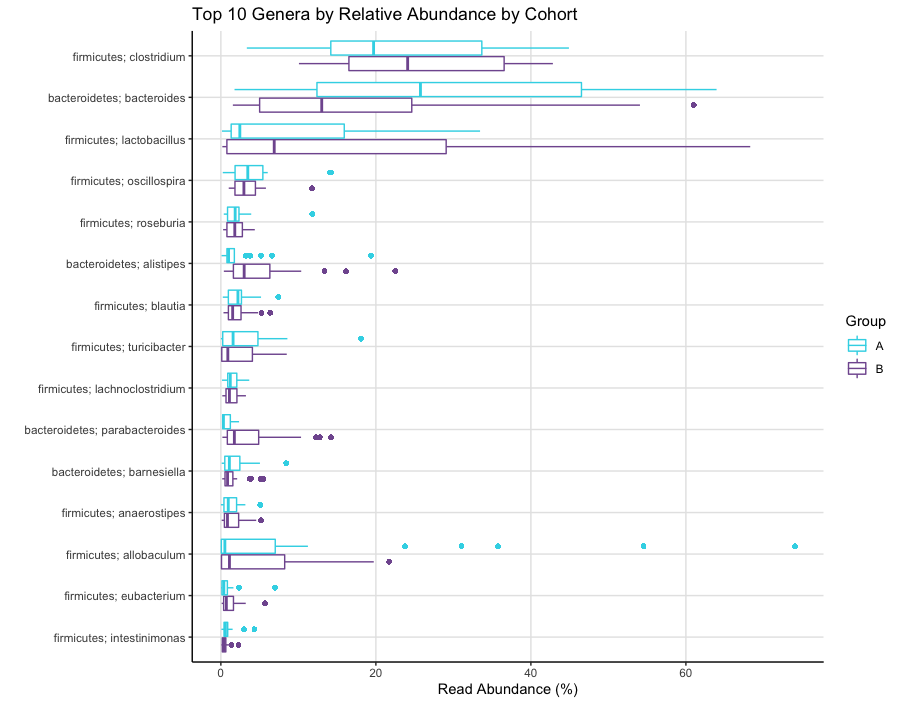

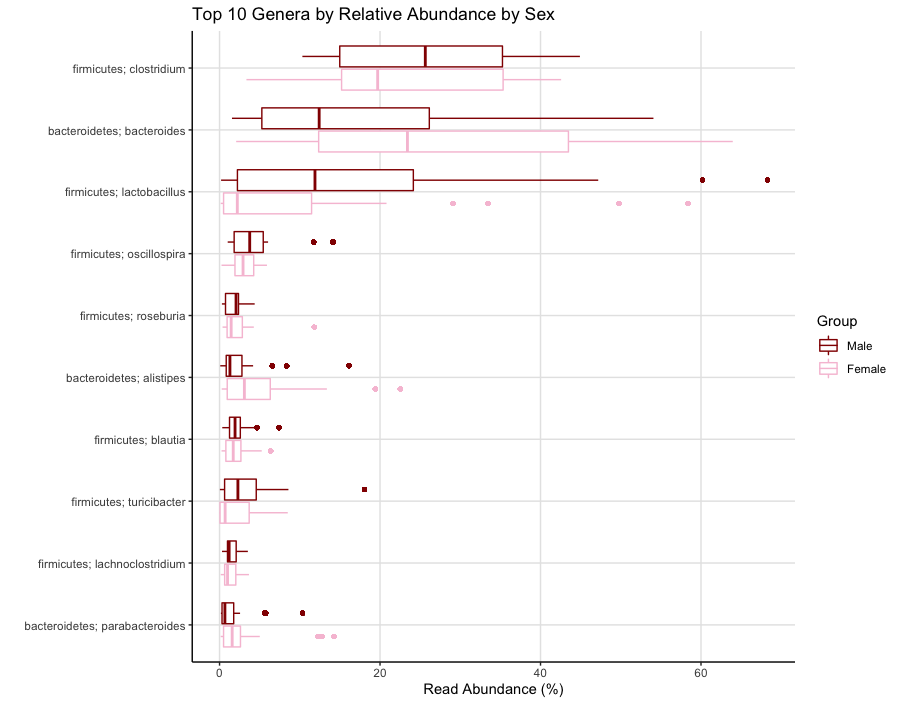

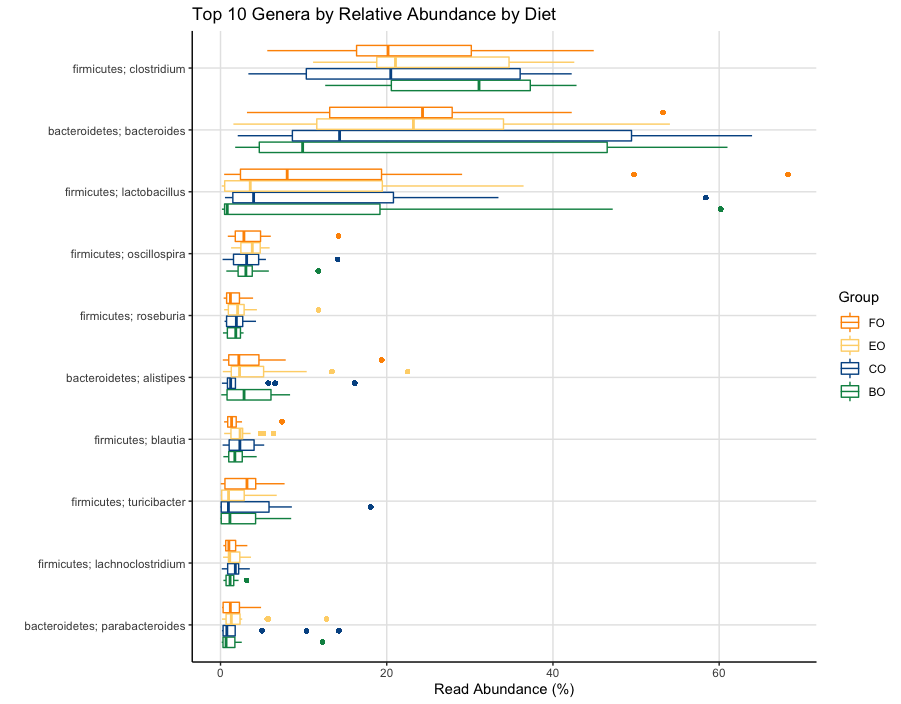


**S1 Fig.** Box and whisker plots of the top 10 most abundant colonic bacterial genera of male and female CD-1® mice received from two different animal husbandry facilities (Cohort A and B, respectively). Mice were fed a high-fat diet either consisting of 100% control fat (CO) or CO fat supplemented with 30% of fish oil (FO), butter oil (BO), or echium oil (EO). Abundance of colonic bacterial genera of mice is displayed by cohort **(A)**, sex **(B)**, and dietary intervention **(C)**.

**
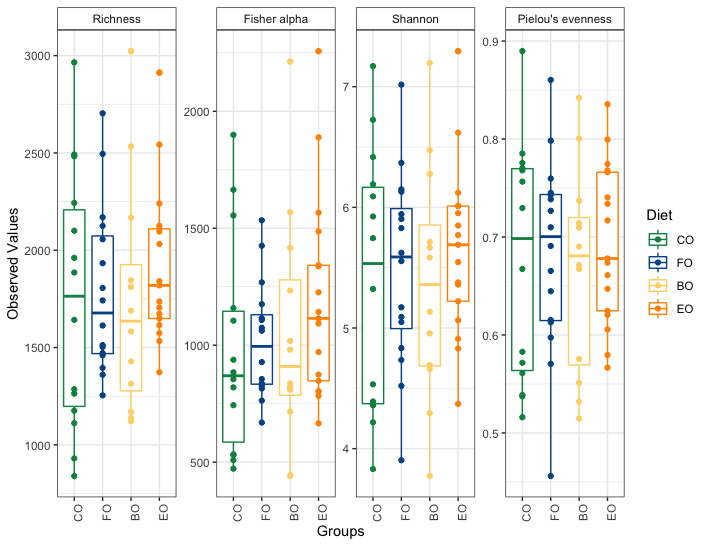

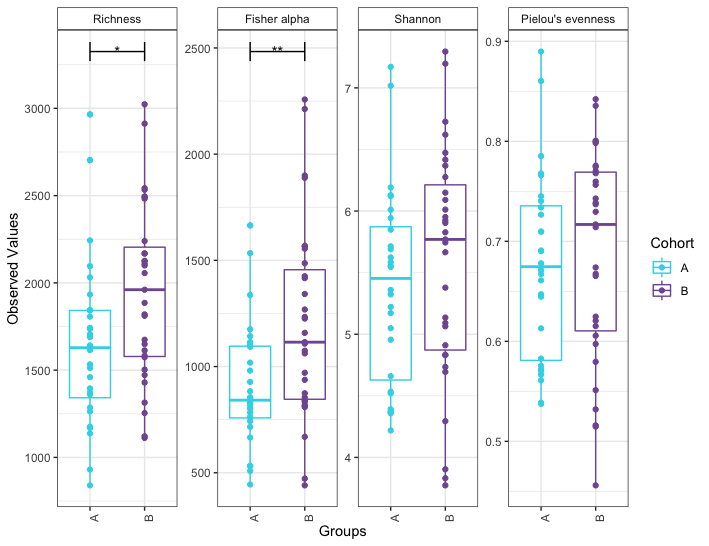

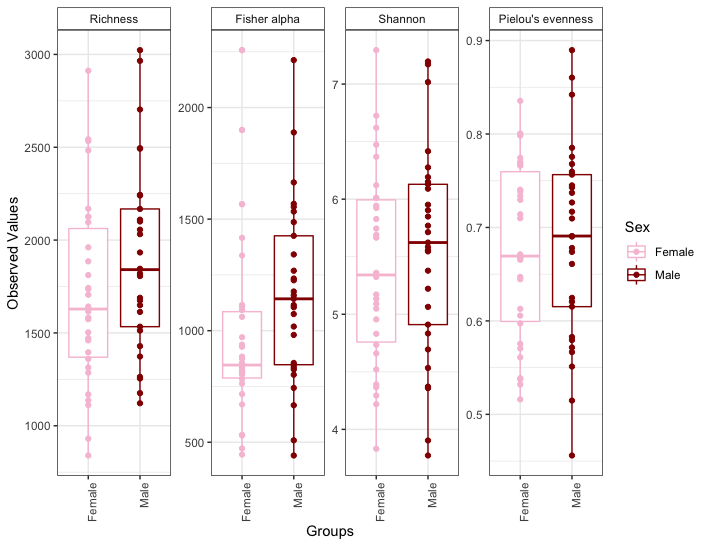
**

**C=C**

**B**

**A**

**S2 Fig.** Alpha diversity indices (richness, Fisher’s alpha, Shannon’s index, Pielou’s evenness) of colonic bacteria of male and female CD-1® mice received from two different animal husbandry facilities (Cohort A and B, respectively). Mice were fed a high-fat diet either consisting of 100% control fat (CO) or CO fat supplemented with 30% of fish oil (FO), butter oil (BO), or echium oil (EO). Alpha diversity indices of colonic bacteria of mice are displayed by cohort **(A)**, sex **(B)**, and dietary intervention **(C)**. ^*^ = *P* < 0.05, ^**^ = *P* < 0.01.

**B**

**A**

**
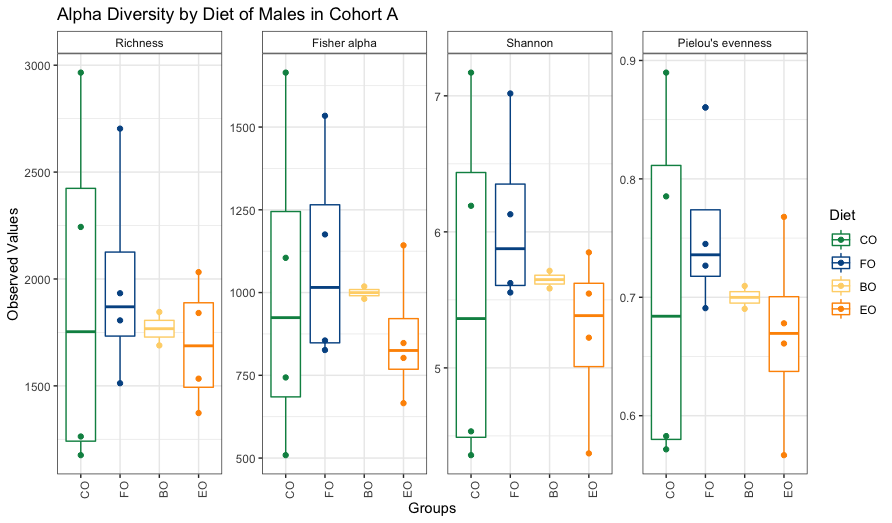
**

**
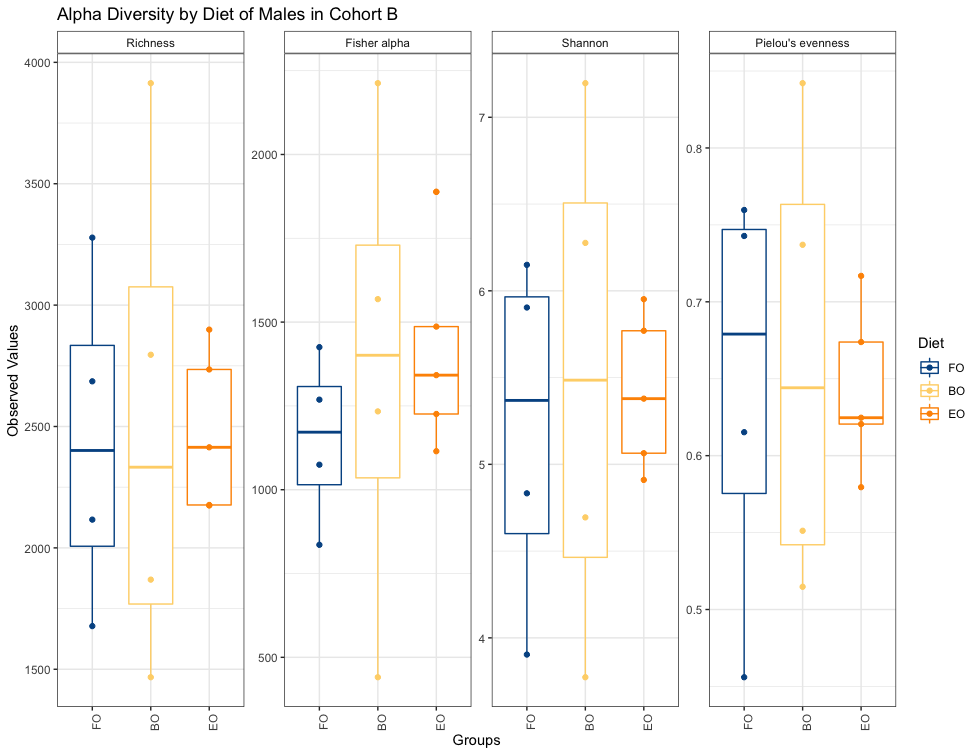
**

**
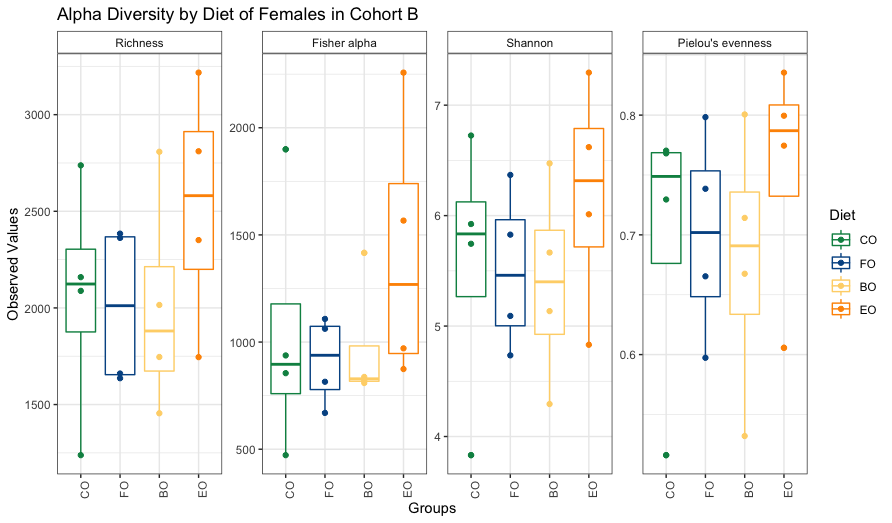

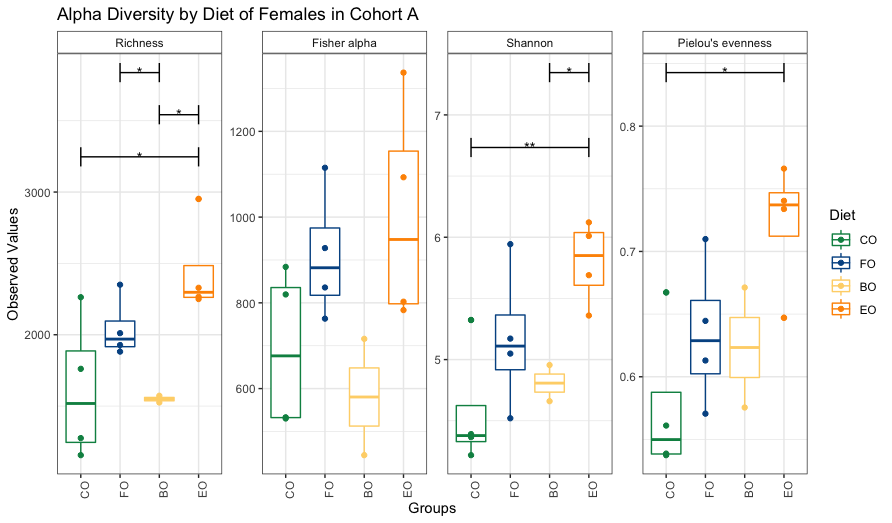
**

**D=C**

**C**

**S3 Fig.** Diet-induced changes in alpha diversity indices (richness, Fisher’s alpha, Shannon’s index, Pielou’s evenness) of colonic bacteria of male and female CD-1® mice received from two different animal husbandry facilities (Cohort A and B, respectively). Mice were fed a high-fat diet either consisting of 100% control fat (CO) or CO fat supplemented with 30% of fish oil (FO), butter oil (BO), or echium oil (EO). Alpha diversity indices of colonic bacteria of mice are displayed for males in Cohort A **(A)**, males in Cohort B **(B)**, females in Cohort A **(C)**, and females in Cohort B **(D)**. ^*^ = *P* < 0.05, ^**^ = *P* < 0.01. CO-fed males from Cohort B were not included in analysis due to insufficient power (n = 2).
